# Die Etablierung eines neuen Forschungskonzepts an einem universitären Standort

**DOI:** 10.1007/s00772-022-00900-6

**Published:** 2022-06-10

**Authors:** Alexandra Gratl, Daniela Lobenwein, Maria Gummerer, Sabine Wipper

**Affiliations:** Univ.-Klinik für Gefäßchirurgie, Medizinische Universität Innsbruck, Anichstraße 35, 6020 Innsbruck, Österreich

**Keywords:** Grundlagenforschung, Translationale Forschung, Lehrforschung, Tierlabor, Forschungsnetzwerk, Basic research, Translational research, Educational research, Animal laboratory, Research network

## Abstract

Neben der klinischen Tätigkeit haben an einem universitären Standort Forschung und Lehre einen großen Stellenwert. Durch die Etablierung eines neuen Forschungslabors an der Universitätsklinik für Gefäßchirurgie der Medizinischen Universität Innsbruck wurden die infrastrukturellen Voraussetzungen zur Gestaltung eines neuen Forschungsschwerpunkts geschaffen. Die Kooperation mit nationalen und internationalen Partnern war für diesen Prozess essenziell. Nicht nur in der Planung und Ausstattung der Räumlichkeiten, sondern auch in der Entwicklung von Studienprotokollen und zur kritischen Diskussion von Ergebnissen ist der Aufbau eines Netzwerkes von großer Bedeutung. Durch die Etablierung der experimentellen Gefäßchirurgie Innsbruck ist nun die Realisierung von Projekten der Grundlagenforschung und der translationalen Forschung an diesem universitären Standort möglich. Zudem spielt die Lehrforschung eine immer größere Rolle, insbesondere um die Ausbildungsstruktur möglichst praxisnah zu gestalten, Nachwuchs anzuwerben und die immer komplexer werdenden Techniken auch praxisnah zu vermitteln.

## Abstract (en)

Apart from daily clinical work and patient care, research and student teaching are essential assignments of a university hospital. The implementation of a completely new research laboratory at the Department of Vascular Surgery at the Medical University of Innsbruck led to the creation of a novel infrastructure for the design and set-up of a newly founded research focus. The cooperation with national as well as international research collaborators was essential for this process. The construction of a research network is not only important for the design and conception of a new research laboratory but also for the development of new study protocols and exchange and discussion of results. After the successful implementation of Experimental Vascular Surgery in Innsbruck, the realization of basic research projects as well as translational research projects is possible at this university location. Moreover, simulation training is an emerging field of research that aims to add more realistic surgical skills to residency programs, attract future residents and teach more complex novel techniques.

## Hinführung zum Thema

An der Universitätsklinik für Gefäßchirurgie der Medizinischen Universität Innsbruck lag der Forschungsschwerpunkt in der Vergangenheit überwiegend im Bereich der klinischen Forschung. Die Grundlagenforschung nahm in den letzten Jahren einen kleinen Stellenwert ein und für die Realisierung von Projekten wurden Kooperationen vor Ort, aber auch auf nationaler und internationaler Ebene etabliert.

Um neue Forschungsschwerpunkte im Bereich der Grundlagenforschung an diesem universitären Standort zu setzen, ist der Aufbau eines experimentellen Forschungslabors essenziell, um ergänzend zu den bereits etablierten und bestehenden Kooperationen und Ressourcen zielgerichtet komplementäre, neue Schwerpunktforschung zu etablieren, die eine weiterführende interdisziplinäre und überregionale Forschung ermöglichen. Des Weiteren kann die Lehrforschung mit Training an Simulatoren die chirurgische Ausbildung restrukturieren und neue Erkenntnisse hervorbringen.

## Strategien zur Etablierung eines Forschungslabors am universitären Standort

Ungeachtet der Art eines Forschungsprojekts (klinische Forschung, translationale Forschung, Grundlagenforschung) ist der Aufbau eines Forschungsnetzwerkes auf nationaler, aber auch auf internationaler Ebene wichtig für den Erfolg des jeweiligen Projektes. Sowohl in der Ideenfindung, der detaillierten Konzipierung eines Projekts, der Patientenrekrutierung als auch in der kritischen Diskussion von Ergebnissen sind Kooperationspartner von großer Bedeutung. Der Aufbau eines überregionalen Forschungsnetzwerks ermöglicht zudem auch eine ressourcenschonende, interdisziplinäre Bearbeitung von Fragestellungen.

Vor allem die Grundlagenforschung ist aus verschiedenen Gründen zumeist an universitären Standorten verortet. Infrastrukturelle, aber vor allem auch personelle Voraussetzungen müssen gegeben sein, um in diesem Bereich Projekte realisieren zu können. Auch wenn die lokalen Voraussetzungen an einer Universitätsklinik im Sinne von Laborräumlichkeiten mit entsprechender Ausstattung gegeben sind, ist der Aufbau eines lokalen Forschungsnetzwerks vor Ort unabdingbar. So können vorhandene Infrastrukturen interdisziplinär genutzt und Forschungsprojekte effektiver, effizienter, kostengünstiger und zeitsparender erarbeitet werden.

Der Aufbau eines Forschungsnetzwerks ist unabdingbar

Insbesondere in der Realisierung von tierexperimentellen Projekten ist die Nutzung von vorhandener Infrastruktur und interdisziplinärer Zusammenarbeit sehr hilfreich. Neben bereits vorhandener Sachkunde für den Umgang mit Tieren ist auch der Erfahrungsaustausch mit anderen Forschungsgruppen in der Etablierung neuer Tiermodelle elementar für den Erfolg eines Projekts. Im Bereich der Großtierexperimente können durch eine interdisziplinäre Projektplanung und Durchführung mehrere Fragestellungen in einem Experiment gebündelt und bearbeitet werden. Dieses Vorgehen ist sowohl ressourcenschonend als auch im Sinne der Tierethik.

An der Universitätsklinik für Gefäßchirurgie der Medizinischen Universität Innsbruck erfolgten zuletzt sämtliche grundlagenorientierte Forschungsprojekte in Kooperation mit lokalen Partnern, da keine abteilungsinterne Infrastruktur vorhanden war. Als Beispiel erfolgte anhand einer engen Kooperation mit der Universitätsklinik für Viszeral‑, Transplant- und Thoraxchirurgie die Ausarbeitung einer Fragestellung sowie die Konzipierung einer tierexperimentellen Studie zum Thema spinale Ischämie. Durch die Möglichkeit der Nutzung der Infrastruktur des dazugehörigen Forschungslabors (Daniel Swarovski Labor) konnte ein Kleintiermodell zur Induzierung einer spinalen Ischämie etabliert werden. Die histopathologischen und immunhistochemischen Auswertungen erfolgten über eine Kooperation mit dem Institut für Pathologie, Neuropathologie und Molekularpathologie. Molekularbiologische Fragestellungen wurden über eine Kooperation mit dem Labor der biologischen Chemie erarbeitet. Allein durch die Etablierung dieses lokalen Netzwerks konnten über die letzten Jahre notwendige und äußerst hilfreiche Kontakte für den Aufbau der eigenen Laborflächen geschaffen werden.

Auch internationale Forschungskooperationen sind für die Etablierung neuer Forschungsschwerpunkte am eigenen Standort von großer Bedeutung. Als Beispiel hierfür konnte durch einen Auslandsaufenthalt an der Klinik für Gefäßchirurgie der Berliner Charité das Forschungsnetzwerk überregional erweitert werden. Die hierdurch gewonnenen Erkenntnisse führten in weiterführenden Kooperationen zur Etablierung eines Forschungsschwerpunkts zu mitochondrialen Pathomechanismen bei Gefäßerkrankungen. Auch ein internationales Forschungsnetzwerk mit Hamburg, Leipzig, Regensburg, Oslo, Houston und Sao Paolo zu unterschiedlichen Forschungsprojekten wurde einbezogen, um den Forschungsschwerpunkt zu erweitern. Dabei wurde darauf geachtet, dass die einzelnen Ressourcen der Kooperationspartner optimal genutzt und die noch benötigten Analysen an dem neu etablierten Forschungsstandort installiert wurden.

## Etablierung der experimentellen Gefäßchirurgie am Beispiel der Medizinischen Universität Innsbruck

Durch die Berufung einer neuen Leitung der Universitätsklinik für Gefäßchirurgie an der Medizinischen Universität Innsbruck wurden sowohl vonseiten der Universität als auch vonseiten des Krankenhausträgers (Tirol Kliniken GmbH) die räumlichen und finanziellen Bedingungen geschaffen, um die Entstehung des Forschungslabors der „experimentellen Gefäßchirurgie“ zu ermöglichen. Nach erfolgter Sanierung mussten die Laborräumlichkeiten entsprechend dem Bedarf der durchzuführenden Projekte ausgestattet werden.

Neben der notwendigen Grundausstattung zur eigenständigen Durchführung grundlegender molekularbiologischer Analysen wurde die restliche Auswahl der Gerätschaften an den Bedarf der bereits bestehenden Forschungskooperationen angepasst. Bei der Ausstattung des Labors wurde explizit darauf geachtet, Geräte zu installieren, die entweder bereits in anderen Forschungsgruppen in Verwendung, jedoch zu ausgelastet oder noch nicht etabliert waren. Mit dieser Strategie soll eine möglichst gute Auslastung der Gerätschaften und des Labors im Sinne einer „core facility“ sichergestellt sein und Kooperationen mit benachbarten Disziplinen ermöglicht werden. Hinsichtlich der bereits laufenden und geplanten tierexperimentellen Studien kann an der Medizinischen Universität Innsbruck auf eine gute Zusammenarbeit mit den lokalen Tierhauseinrichtungen zurückgegriffen werden, sodass für diese Projektvorhaben keine zusätzlichen infrastrukturellen Voraussetzungen geschaffen werden müssen.

Um den Aufwand und die Herausforderungen der Neuetablierung eines Forschungslabors personell zu ermöglichen, erfolgten gezielte, zeitlich begrenzte Freistellungen der universitären ärztlichen Mitarbeiter, sodass insbesondere der in der Anfangsphase hohe administrative Aufwand bewältigt werden konnte. Um eine erfolgreiche Durchführung der geplanten Forschungsvorhaben zu ermöglichen, wurde einerseits eine Postdoc-Stelle etabliert, andererseits soll der Personalsatz durch projektspezifische Drittmittelanträge langfristig erhöht werden. Zusätzlich stehen studentische Mitarbeiter dem vorhandenen universitären Personal zur Verfügung und leisten einen wesentlichen Beitrag zur Realisierung der Projektvorhaben. Entsprechend der österreichischen Ausbildungsordnung ist es auch möglich, im Rahmen der Weiterbildung zum Gefäßchirurgen ein „Wissenschaftsmodul“ über einen Zeitraum von 9 Monaten zu absolvieren, sodass auch hier eine gezielte Förderung von motivierten und an der Grundlagenforschung interessierten jungen KollegInnen ermöglicht werden kann.

## Entwicklung eines Forschungskonzepts

**Figure 1:**
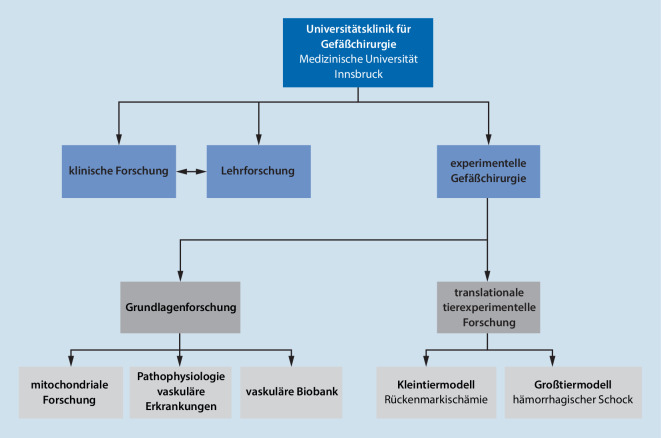

Das Forschungskonzept an der experimentellen Gefäßchirurgie Innsbruck wurde Anhand bestehender nationaler und internationaler Kooperationen und Erfahrungen strukturiert. Eine grafische Darstellung erfolgt in Abb. 1:

### Grundlagenforschung

#### Schwerpunkt mitochondriale Forschung

Bei einer chronischen Minderperfusion durch flusslimitierende arterielle Stenosen kommt es in betroffenen Muskelabschnitten zu histomorphologischen und metabolischen Veränderungen [8, 17, 18]. Eine entscheidende Rolle in diesem pathophysiologischen Prozess spielen hierbei Mitochondrien [15].

Mitochondrien spielen eine wesentliche pathophysiologische Rolle

Zur Untersuchung der Mitochondrienfunktion stehen verschiedene Methoden sowohl in vivo als auch in vitro zur Verfügung, wobei die hochauflösende Respirometrie durch Untersuchung der verschiedenen Komplexe der Atmungskette unter kontrollierten biochemischen Bedingungen den „golden standard“ darstellt [7]. An der Klinik für Gefäßchirurgie der Charité Berlin wurde der Effekt einer offen chirurgischen oder endovaskulären Revaskularisation bei Stenosen oder Verschlüssen der A. femoralis superficialis auf die Mitochondrienfunktion untersucht. Anhand dieser Studien konnte gezeigt werden, dass Mitochondrien in der Lage sind, ihre präoperativ eingeschränkte Funktion nach Wiederherstellung des Blutflusses zu regenerieren [6]. Nicht betroffene Muskelabschnitte zeigten keine Veränderungen nach erfolgter Therapie, sodass hier der Rückschluss gezogen wurde, dass die Revaskularisation an sich einen bedeutenderen Effekt auf die Mitochondrienfunktion im Vergleich zu einer gesteigerten körperlichen Aktivität hat [7]. Anhand der Vorergebnisse sollen nun in Kooperation mit der Berliner Charité Folgeprojekte an der experimentellen Gefäßchirurgie Innsbruck durchgeführt werden. Zunächst ist die Durchführung einer randomisierten kontrollierten Studie – das MIVA-Trial – mit dem Ziel der Evaluierung der Auswirkungen verschiedener Behandlungsstrategien (konservative Therapie mittels Gehtraining versus interventionelle Revaskularisation) auf die Mitochondrienfunktion geplant. Des Weiteren sollen klinisch gut anwendbare Methoden zur Untersuchung der distalen Perfusion (Oszillographie, Knöchel-Arm-Index, NIRS, Hyperspektralkamera jeweils vor und nach körperlicher Belastung) mit den Ergebnissen der hochauflösenden Respirometrie korreliert und so ein translationaler Aspekt der Untersuchungen ermöglicht werden. Entsprechend der Forschungsschwerpunkte des Standorts wurde daher ein Oxygraph 2k Series I (Oroboros Instruments, Innsbruck, Österreich) zur Durchführung der hochauflösenden Respirometrie erworben. Dieser ermöglicht zukünftig weitreichende sowohl translationale als auch grundlagenwissenschaftliche Forschungsprojekte und stellt in interdisziplinären Projekten eine Basis für die Erarbeitung spezifischer gefäßchirurgischer Fragestellungen dar. Ferner wurde eine Postdoc-Stelle mit fachlicher Qualifikation auf diesem Themengebiet eingerichtet.

#### Schwerpunkt Pathophysiologie vaskulärer Erkrankungen

Ein weiterer Forschungsschwerpunkt der experimentellen Gefäßchirurgie Innsbruck liegt in der Erforschung zellulärer sowie subzellulärer pathophysiologischer Mechanismen vaskulärer Erkrankungen wie den Aortenpathologien oder der peripheren arteriellen Verschlusskrankheit.

Aortenpathologien, insbesondere akute Aortensyndrome, sind eine medizinische Entität mit einer hohen Morbidität und Mortalität [16]. Zudem zeigt sich in der klinischen Praxis, dass auch PatientInnen, die aufgrund des Aortendiameters noch keine Behandlungsindikation ihres Aortenaneurysmas hatten, mit akuten Aortensyndromen wie einer Aortenruptur oder Aortendissektion klinisch vorstellig werden [5, 11].

Aortensyndrome haben eine hohe Morbidität und Mortalität

Es wird angenommen, dass glatte Gefäßmuskelzellen („vascular smooth muscle cells“, VSMCs), die in der Media der Aortenwand zu finden sind, auf zellulärer Ebene hauptsächlich an der Entstehung von Aortenpathologien beteiligt sind [1]. Eine Degradierung der extrazellulären Matrixproteine der Aortenwand trägt zu einer zusätzlichen mechanischen Schwächung der Aortenwand bei [1]. Allerdings ist bis dato noch sehr wenig über die exakte Pathophysiologie dieser Erkrankungen bekannt. Deshalb ist die Erforschung sowohl der aortalen Pathophysiologie als auch möglicher Einflussfaktoren, u. a. die mechanische Belastung der Aorta auf die zelluläre Homöostase, ein Schwerpunkt unseres Zellkulturallabors. Mithilfe eines neu erworbenen Flexcell FX6000^TM^ Tension System sowie des Flexcell Streamer^TM^ System können sowohl mechanische Belastungen als auch „fluid shear stress“ (Belastung der Gefäßwand bedingt durch Veränderungen im Blutfluss aufgrund von pathologischen Veränderungen wie zum Beispiel im Rahmen der Flussveränderungen bei aneurysmatischen Erkrankungen) in vitro unter standardisierten Bedingungen erforscht werden. Ein Ziel dieses Forschungsschwerpunkts ist es, in Zusammenschau mit klinischen Daten eine neue, personenspezifische Risikostratifizierung des Rupturrisikos zu erarbeiten, sowie ein besseres Verständnis der Pathophysiologie von Aortensyndromen.

Im Zusammenhang mit der Erforschung der vaskulären mechanischen Belastung und dem „fluid shear stress“ spielt auch die genauere Erforschung der peripheren arteriellen Verschlusskrankheit (PAVK) eine große Rolle. Nach erfolgreicher interventioneller sowie operativer Revaskularisation von flusslimitierenden arteriosklerotischen Stenosen und Verschlüssen bei PatientInnen mit PAVK kommt es oftmals trotz optimaler medikamentöser Therapie zu Restenosen oder Verschlüssen der gefäßchirurgischen Rekonstruktionen. Aus hämodynamischer Sicht kommen Flussbeschleunigungen am proximalen oder distalen Anschlusspunkt durch eine Intimahyperplasie und damit einhergehend eine erhöhte mechanische Belastung der Gefäßwand als Ursache dieser Komplikationen infrage [13]. Ziel dieses Forschungsschwerpunkts ist es, mögliche Einflüsse mechanischer Belastung im Sinne von Flussbeschleunigungen auf die zelluläre Homöostase betroffener Gefäßwandzellen (Endothelzellen, VSMCs, Fibroblasten) zu untersuchen und so mögliche neue Erkenntnisse und ggf. Behandlungsmethoden für Rezidivstenosen- oder Verschlüsse zu entwickeln. Durch die Anschaffung des Flexcell FX6000^TM^ Tension System sowie des Flexcell Streamer^TM^ System konnte die Basis für zahlreiche lokale Kooperationen geschaffen werden, um die Geräte im Sinne einer „core facility“ nutzen zu können.

Insbesondere in Verbindung mit der neu etablierten Biobank könnten neue Erkenntnisse in diesem Bereich zu einem verbesserten Behandlungsergebnis bei PAVK beitragen.

#### Etablierung einer vaskulären Biobank

Wie bereits durch frühere Kohortenstudien festgestellt, zählen zunehmendes Alter, Rauchen, Hypertonie, Hypercholesterinämie und Diabetes mellitus zu den klassischen Risikofaktoren für die Entstehung insbesondere atherosklerotischer vaskulärer Erkrankungen [14]. Dem entgegensetzt geht man vor allem im Bereich aneurysmatischer Gefäßerkrankungen von einer bedeutenden genetischen Prädisposition aus [2–4]. Eine genaue Bestimmung der Risikofaktoren für Aortenerkrankungen, abseits von genetischen Syndromen wie etwa Marfan Syndrom, ist – insbesondere auch aufgrund der Inhomogenität des Patientenkollektivs – deutlich erschwert. Im klinischen Alltag zeigt sich jedoch, dass PatientInnen auch abseits der angeführten Risikofaktoren an vaskulären Krankheiten leiden. Zudem ist oftmals zu beobachten, dass es bei PatientInnen trotz optimaler Therapie (medikamentös, interventionell und/oder operativ) zu einem Fortschreiten ihrer vaskulären Erkrankungen kommt, während gleich gelagerte Fälle über Jahre hinweg stabile Krankheitsverläufe ohne wesentliche Progression aufweisen. Somit sind erstere PatientInnen bereits frühzeitig von Komplikationen wie beispielsweise dem Verlust betroffener Extremitäten infolge von Minderperfusion oder von anderen Komplikationen betroffen, während andere PatientInnen mit denselben vaskulären Erkrankungen über Jahre hinweg einen stabilen Krankheitsverlauf ohne wesentliche Progression aufweisen.

Daher ist ein weiterer Forschungsschwerpunkt der experimentellen Gefäßchirurgie Innsbruck die Etablierung eines umfassenden klinischen Registers sowie einer Biobank von PatientInnen mit vaskulären Erkrankungen. Das klinische Register dient zunächst einer besseren Charakterisierung einer umfangreichen Patientenpopulation. Gemessen am aktuellen Stand der Wissenschaft ist eine prospektive Erfassung und Auswertung klinischer Daten im Rahmen einer Registerstudie dafür erste Wahl. In weiterer Folge sollen relevante Unterschiede innerhalb der Kohorte, beispielsweise zwischen PatientInnen mit progredienten und stabilen Verläufen einer PAVK, analysiert werden. Die Ergebnisse könnten somit beispielsweise der optimierten, personenbezogenen Risikostratifizierung dienen und damit möglicherweise auch zu künftigen Therapieentscheidungen beitragen.

Etablierung eines klinischen Registers und einer vaskulären Biobank

Neben dem Aufbau eines klinischen Registers ist auch die Etablierung einer Biobank von Blut‑, Harn‑, Liquor- und Gewebsproben der rekrutierten PatientInnen geplant. Dies wird in Kooperation mit der zentralen Biobank der Medizinischen Universität Innsbruck unter der Leitung des Instituts für Pathologie, Neuropathologie und Molekularpathologie realisiert.

Diese Biobank wird sowohl der langfristigen Aufbewahrung als auch Aufarbeitung und Analyse der entnommenen Proben in unterschiedlichen Versuchsanordnungen dienen. Dazu gehören u. a. auch die Biomarker, die in der Diagnostik und Verlaufsprogression kardialer Erkrankungen eine breite Anwendung gefunden haben. Während das Ausmaß und Vorhandensein einer Myokardschädigung klinisch beispielsweise anhand der Serum-Kreatinkinase sowie der Serum-Troponine detektiert und quantifiziert werden kann, existieren derzeit keine validen Biomarker, weder zur Detektion noch zur Verlaufsbeurteilung, für andere vaskuläre Erkrankungen. Gerade für PatientInnen mit Aortenaneurysmen stellt diese diagnostische Lücke im klinischen Alltag erhebliches Problem dar, da 95 % der betroffenen PatientInnen initial klinisch symptomfrei sind [10]. Neben der Evaluierung neuer Biomarker ist es langfristiges Ziel der vorgestellten Studie, vaskuläre Erkrankungen auf zellulärer und biochemischer Ebene besser zu verstehen, und damit Ansätze für die Entwicklung neuer Therapien zur Verfügung stellen zu können (s. oben).

### Tierexperimenteller grundlagenwissenschaftlicher und translationaler Forschungsschwerpunkt

In-vivo-Tiermodelle stellen sowohl im Bereich der Grundlagenwissenschaften als auch der translationalen Forschung eine entscheidende Säule der Forschungsarbeit dar. Sie sind daher an einer universitären Einrichtung unverzichtbar. Vor allem der interdisziplinäre nationale und internationale Austausch, beginnend in der Projektplanungsphase bis hin zur Durchführung, ist im Rahmen tierexperimenteller Versuche entscheidend. So können nicht nur die vorhandener Infrastrukturen besser ausgelastet, sondern auch mehrere Fragestellungen in einem Experiment gleichzeitig bearbeitet werden. Sowohl im Sinne einer effizienten Verwendung bestehender Ressourcen als auch im Sinne tierethischer Bedenken sind diese Überlegungen entscheidend für den Erfolg tierexperimenteller Forschungsprojekte.

Durch lokale Kooperationen mit der Universitätsklinik für Viszeral‑, Thorax- und Transplantchirurgie wurde bereits ein Kleintiermodell zur Induzierung einer spinalen Ischämie etabliert. Hierfür wird mittels Ballonokklusion der thorakalen Aorta temporär die Rückenmarksperfusion unterbrochen und eine Klemmung der Aorta simuliert [9]. Anhand dieses Modells erfolgt derzeit die Austestung des neuroprotektiven Potenzials von Tetrahydrobiopterin (BH4), einem essenziellen Kofaktor der NO-Synthase. BH4 ist entscheidend in der Bildung von NO. Ein Verlust des intrazellulären BH4 führt über eine Entkoppelung der NO-Synthase zu einer vermehrten Sauerstoffradikalbildung [19, 21].

Tiermodelle sind essenziell für die Beantwortung spezifischer Fragestellungen

Durch Kooperation mit dem Universitären Herz- und Gefäßzentrum Hamburg konnte ein Großtiermodell zur Induktion eines hämorrhagischen Schocks etabliert und mit Unterstützung der Klinik für Anästhesie in Innsbruck implementiert werden. Anhand dieses Models erfolgt die Austestung eines neuartigen auf Polyphosphat basierenden Hämostyptikums, dessen Effektivität mit einem bisher herkömmlichen Hämostyptikum (CombatGauze®) verglichen wird. Gleichzeitig werden die Auswirkungen verschiedener Beatmungsformen im hämorrhagischen Schock von der Universitätsklinik für Anästhesie evaluiert. Vor allem der interdisziplinäre nationale und internationale Austausch von der Projektplanungsphase bis hin zur Durchführung tierexperimenteller Versuche ist entscheidend. So können nicht nur vorhandene Infrastrukturen besser ausgelastet, sondern auch mehrere Fragestellungen in einem Experiment gleichzeitig bearbeitet werden. Eine effiziente Verwendung der Ressourcen, auch zur Vermeidung tierethischer Bedenken, sind für den Erfolg tierexperimenteller Forschungsprojekte mitentscheidend.

### Lehrforschung

Neben ärztlichen Tätigkeiten in der Patientenversorgung und Forschung, ist die (studentische) Lehre eine der Hauptaufgaben einer universitären Klinik. Des Weiteren gewinnt in den letzten Jahren aufgrund von signifikanten Änderungen der postpromotionellen Ausbildung die strukturierte Ausbildung in den jeweiligen Fachgebieten zunehmend an Bedeutung. Insbesondere an universitären Standorten nimmt nicht nur die Komplexität spezifischer Eingriffe zu, sondern auch der Grad an Spezialisierungen. In chirurgischen Fächern wird derzeit aufgrund der Covid-19-Pandemie und der damit einhergehenden reduzierten Anzahl an Interventionen und chirurgischen Eingriffen ein Training am Simulator zu Ausbildungszwecken immer interessanter [12, 20].

Das Üben und Erlernen von chirurgischen und endovaskulären Fertigkeiten am Modell ermöglicht die schrittweise Aneignung chirurgischer Prozeduren und der Auszubildende bekommt Routine im Umgang mit verschiedenen Materialien und Vorgehensweisen. Es kommt somit zu einer optimierten Ausbildung junger ÄrztInnen und StudentInnen, verbunden mit einer deutlichen Risikoreduktion für PatientInnen. Somit ist die Etablierung eines geförderten Trainings an Simulationsmodellen mit anschließender Evaluation und eine damit verbundene Etablierung eines strukturierten chirurgischen Ausbildungssystems vielversprechend.

Im Zuge der Neustrukturierung der Forschungsschwerpunkte wurde an der Universitätsklinik für Gefäßchirurgie die Lehrforschung als weiterer Forschungsschwerpunkt etabliert. Dieser ermöglicht unter anderem die Erforschung des bestmöglichen strukturierten Trainings am Simulator. Um Erfahrungen auszutauschen und Hilfestellungen in der Etablierung des Studienprotokolls zu bekommen, wird auf eine internationale Kooperation mit den Universitätskliniken für Gefäßchirurgie von Sao Paulo und Hamburg zurückgegriffen. Lokal besteht eine Kooperation mit der Universitätsklinik für Radiologie der Medizinischen Universität Innsbruck. Mithilfe von freiwillig teilnehmenden Studenten werden verschiedener Trainingsmodelle erprobt, die Trainingsfortschritte analysiert und Überprüfungskonzepte evaluiert.

Die Entwicklung eines optimalen, strukturierten Ausbildungsplans, der auch Simulatortrainings in entsprechenden Abständen umfasst, mit daraus resultierender Erhöhung der Patientensicherheit, ist ein Hauptziel der Lehrforschungsgruppe der Universitätsklinik für Gefäßchirurgie der Medizinischen Universität Innsbruck.

Ein weiteres Ziel der Lehrforschung besteht darin, neue Strategien zur Rekrutierung neuer ärztlicher Mitarbeiter zu erarbeiten. Während des Medizinstudiums ist das Fach Gefäßchirurgie sowohl in der Theorie als auch in der Praxis häufig unterrepräsentiert und kommt vielen Studenten als Berufswunsch nicht in den Sinn. Es ist deshalb von großer Bedeutung, schon früh mit Studenten in Kontakt zu treten und die Gefäßchirurgie als eigenständiges Fach attraktiv zu machen. Mittels einer Fragebogenstudie wurde der Einfluss von chirurgischen Praktika auf die spätere Berufsauswahl analysiert. Dies erfolgt an unserem Standort in enger Kooperation mit der Universitätsklinik für Viszeral‑, Transplantations- und Thoraxchirurgie sowie dem Institut für klinisch-funktionelle Anatomie. Anhand dieser Befragung von Studierenden konnte bestätigt werden, dass viele Studenten in ihrer Ausbildung häufig Berührungspunkte mit der Viszeralchirurgie hatten, jedoch sehr selten mit Gefäßchirurgie.

## Zusammenfassung und Ausblick

Durch die Etablierung der experimentellen Gefäßchirurgie Innsbruck konnten die infrastrukturellen Voraussetzungen zur Festigung eines neuen, breit aufgestellten Forschungsschwerpunkts (Grundlagenforschung, translationale Forschung, Lehrforschung) an der Universitätsklinik für Gefäßchirurgie geschaffen werden. Im Prozess der Implementierung eines neuen Forschungslabors sind sowohl nationale als auch internationale Kooperationspartner und Forschungsnetzwerke elementar. Ein universitäres Umfeld ist neben dem wissenschaftlichen Austausch auch zur Schaffung der personellen Voraussetzungen unerlässlich. Hilfestellungen von Partnern sind in allen Bereichen, beginnend bei der Planung der Räumlichkeiten bis hin zu spezifischen projektbezogenen Fragestellungen, von großer Bedeutung, um Probleme frühzeitig zu erkennen und beseitigen zu können. Ferner können sich durch Kooperationen und den interdisziplinären wissenschaftlichen Austausch neue Fragestellungen, neue wissenschaftliche Ansätze und Synergien in der Erforschung und ergeben.

## Fazit für die Praxis

- Die weitere Erforschung von pathophysiologischen Abläufen in der Entstehung von Gefäßerkrankungen ist für das Verständnis, aber auch für die Entwicklung neuer Therapieansätze von großer Wichtigkeit.
- Der Aufbau eines Forschungsschwerpunkts an einem universitären Standort bietet sowohl die forschungsspezifischen als auch die klinischen Voraussetzungen, um erfolgreiche Projekte durchführen zu können.
- Insbesondere in translationalen Forschungsprojekten ist zur erfolgreichen Patientenrekrutierung eine klinische Anbindung notwendig.
- Eine enge Zusammenarbeit des klinischen und des wissenschaftlichen Personals ist zur Abwicklung dieser Projekte wichtig.
- Die Implementierung von Projekten im Bereich der Lehrforschung ist ein wichtiger Baustein, um die chirurgische Ausbildung zu optimieren.

## References

[1] Ailawadi G, Eliason JL, Upchurch GR (2003). Current concepts in the pathogenesis of abdominal aortic aneurysm. J Vasc Surg.

[2] Albornoz G, Coady MA, Roberts M (2006). Familial thoracic aortic aneurysms and dissections—incidence, modes of inheritance, and phenotypic patterns. Ann Thorac Surg.

[3] Booher AM, Eagle KA (2011). Diagnosis and management issues in thoracic aortic aneurysm. Am Heart J.

[4] Braverman AC (2015). Heritable thoracic aortic aneurysm disease: recognizing phenotypes, exploring genotypes. J Am Coll Cardiol.

[5] Elefteriades JA, Farkas EA (2010). Thoracic aortic aneurysm: clinically pertinent controversies and uncertainties. J Am Coll Cardiol.

[6] Gratl A, Frese J, Speichinger F (2019). Regeneration of mitochondrial function in gastrocnemius muscle in peripheral arterial disease after successful Revascularisation. Eur J Vasc Endovasc Surg.

[7] Gratl A, Wipper S, Frese JP (2021). The role of mitochondrial function in peripheral arterial disease: insights from translational studies. Int J Mol Sci.

[8] Hiatt WR, Nawaz D, Brass EP (1987). Carnitine metabolism during exercise in patients with peripheral vascular disease. J Appl Physiol.

[9] Hwang JY, Sohn HM, Kim JH (2017). Reproducible motor deficit following aortic occlusion in a rat model of spinal cord ischemia. J Vis Exp.

[10] Isselbacher EM (2005). Thoracic and abdominal aortic aneurysms. Circulation.

[11] Kim JB, Spotnitz M, Lindsay ME (2016). Risk of aortic dissection in the moderately dilated ascending aorta. J Am Coll Cardiol.

[12] Klass D, Tam MD, Cockburn J (2008). Training on a vascular interventional simulator: an observational study. Eur Radiol.

[13] Kleinstreuer C, Hyun S, Buchanan JR (2001). Hemodynamic parameters and early intimal thickening in branching blood vessels. Crit Rev Biomed Eng.

[14] Laslett LJ, Alagona P, Clark BA (2012). The worldwide environment of cardiovascular disease: prevalence, diagnosis, therapy, and policy issues: a report from the American college of cardiology. J Am Coll Cardiol.

[15] Makris KI, Nella AA, Zhu Z (2007). Mitochondriopathy of peripheral arterial disease. Vascular.

[16] Olsson C, Thelin S, Stahle E (2006). Thoracic aortic aneurysm and dissection: increasing prevalence and improved outcomes reported in a nationwide population-based study of more than 14,000 cases from 1987 to 2002. Circulation.

[17] Pipinos I, Judge AR, Selsby JT (2007). The myopathy of peripheral arterial occlusive disease: part 1. Functional and histomorphological changes and evidence for mitochondrial dysfunction. Vasc Endovascular Surg.

[18] Pipinos I, Judge AR, Selsby JT (2008). The myopathy of peripheral arterial occlusive disease: part 2. oxidative stress, neuropathy, and shift in muscle fiber type. Vasc Endovascular Surg.

[19] Pou S, Pou WS, Bredt DS (1992). Generation of superoxide by purified brain nitric oxide synthase. J Biol Chem.

[20] See KW, Chui KH, Chan WH (2016). Evidence for endovascular simulation training: a systematic review. Eur J Vasc Endovasc Surg.

[21] Werner ER, Gorren AC, Heller R (2003). Tetrahydrobiopterin and nitric oxide: mechanistic and pharmacological aspects. Exp Biol Med.

